# The Importance of a Participatory and Integrated One Health Approach for Rabies Control: The Case of N’Djaména, Chad

**DOI:** 10.3390/tropicalmed2030043

**Published:** 2017-08-23

**Authors:** Monique Lechenne, Rolande Mindekem, Séraphin Madjadinan, Assandi Oussiguéré, Daugla Doumagoum Moto, Kemdongarti Naissengar, Jakob Zinsstag

**Affiliations:** 1 Swiss Tropical and Public Health Institute, P.O.Box, Socinstrasse 57, CH-4002 Basel, Switzerland; jakob.zinsstag@unibas.ch; 2 University of Basel, Petersplatz 1, CH-4051 Basel, Switzerland; 3 Centre de Support en Santé International, BP: 972, Moursal, N’Djaména, Chad; mrola2002@yahoo.fr (R.M.); mseraphin41@gmail.com (S.M.); daugla.doumagoum@gmail.com (D.D.M.); 4 Institut de Recherché en Elevage pour le Développement, BP: 433, Farcha, N’Djaména, Chad; assandi_oussiguere@yahoo.fr (A.O.); naissengar@gmail.com (K.N.)

**Keywords:** rabies incidence, post-exposure prophylaxis, integrated bite case management (IBCM), One Health

## Abstract

This study compares data on animal rabies cases from the Chadian national rabies laboratory, hosted at the Insitut de Recherche en Elevage pour le Developpement (IRED), with bite case reporting from health facilities. The data collection accompanied a mass dog vaccination intervention over two years in N’Djaména, Chad. This allowed for a comparison of the dynamics of the incidence of animal rabies cases, human bite exposure incidence and post-exposure prophylaxis (PEP) demand during a dog rabies elimination attempt. Following the mass vaccination, the monthly animal rabies incidence dropped from 1.1/10,000 dogs, as observed prior to the campaign in 2012, to 0.061/10,000 dogs in 2014. However, the PEP demand was found to be largely unaffected. The suspicion of the rabies exposure as reported by health personnel in most cases did not reflect the status of the biting animal but rather the severity of the bite wound, resulting in inappropriate PEP recommendations. In addition, the levels of reporting dead or killed animals to the rabies laboratory was found to be very low. These results reveal a profound lack of communication between health facilities and veterinary structures and the absence of an integrated bite case management (IBCM) approach. Improved communication between human health and veterinary workers is imperative to prevent human rabies deaths through the appropriate use of PEP and to further translate success in animal rabies control into cost savings for the public health sector through a lower PEP demand. Improved training of health and veterinary personnel and the sensitisation of the public are needed to achieve good IBCM practice, to increase the rate of diagnostic testing, to provide adequate and timely PEP, and to reduce the wastage of scarce vaccine resources.

## 1. Introduction

Within a One Health framework, rabies is likely the best documented example for the added value of closer collaboration of human and veterinary medicine for the control of zoonotic diseases [1]. With a few singular exceptions, humans only contract the disease through contact with an infected animal [2]. The highest disease burden is found in resource-poor settings where rabies is endemic in domestic dogs, which are the predominant species causing exposure in humans [3]. Poverty negatively impacts the access to post-exposure prophylaxis (PEP), which is urgently needed after a bite from a suspected rabid animal. In most rabies-endemic countries in Africa, the cost for PEP exceeds the monthly income of people living below the poverty level [1,3,4]. In many remote areas, PEP is not available because of long distances to a health facility or the inefficiency of the health system [5]. In order to achieve the challenging goal of zero human deaths from dog-mediated rabies by 2030, as postulated by the World Health Organization (WHO) and partners [6], access to and the adequate use of PEP must be implemented. Because only a fraction of all bite cases are inflicted by a rabid animal [7,8] and because many countries face a shortage of the human vaccine for PEP, an integrated bite case management (IBCM) approach is required to guide treatment recommendations in order to save the highest percentage of human lives in the short term. The long-term sustainable control of rabies and the reduction of human fatalities can only be achieved through interventions that interrupt transmission in the reservoir species. Vaccination of dogs is the only control measure that will lead to the elimination of rabies in domestic animals and result in a reduction of the exposure risk in humans by more than 90% [3]. Dog vaccination reduces the need for PEP and considerably reduces the burden of premature deaths from rabies, averting a high number of years of life lost (YLL) [9,10]. Therefore, investment in dog vaccination, especially mass vaccination, although potentially more expensive than prevention in humans in the short term, is advantageous in the long term, with higher cost-efficiency compared to the cumulative costs of PEP alone [11,12,13]. However, reduction of the dog rabies incidence does not necessarily translate directly into reduced demand for PEP. Rabies control can lead to even higher PEP demand in the face of decreasing the exposure risk, which can be explained by heightened rabies awareness in the community [14,15,16]. To maximise the beneficial financial effects, dog vaccination should be carried out in conjunction with IBCM to prevent the overuse of PEP. The identification of bite victims who are not exposed to a suspected animal can be achieved through closer communication of medical staff with veterinary workers who are informed of the status of the respective animal. Ultimately, IBCM would also improve surveillance for the validation (proof of the absence of dog-mediated human rabies deaths) and verification (proof of the absence of dog rabies) of the 2030 goal.

The present study describes a lack of communication between the human and animal health sectors in the absence of IBCM, as shown by the comparison of laboratory and health center data. The data collection was performed during an epidemiological follow up study on the dynamics of the dog rabies incidence, the human dog-bite incidence and the PEP demand during a rabies elimination program in N’Djaména, Chad.

## 2. Methods

### 2.1. Background

In 2000, research on rabies control was initiated in N’Djaména, the capital city of Chad. Prior to the intervention, rabies was endemic in the local dog population, circulating at a low and stable level with an effective reproductive ratio (R_e_) of just over 1 [12]. Pilot vaccination campaigns in 2003 and 2006 validated the feasibility of dog vaccination in the city, showing good participation by dog owners, provided the vaccine was offered without charge [17,18].

Given the promising initial results, the Swiss Tropical and Public Health Institute (Swiss TPH) implemented a large-scale mass dog vaccination intervention, together with two local partners, the Institut de Recherche en Elevage pour le Développement (IRED) and the Centre de Support en Santé International (CSSI). In May 2012, community awareness was emphasised to obtain accurate incidence data before the planned vaccination campaigns. Posters in French and Arabic illustrating the best practices after a bite incident were distributed to health centers, hospitals, pharmacies and veterinary facilities throughout N’Djamena. Drawings were used to accompany the text, because of the high illiteracy rate in Chad. The information included the importance of washing wounds after a bite, the need to seek medical treatment, and the importance of contacting a veterinarian (in the case of a live animal) or bringing the body to the IRED (in the case of animal death). Fixed-post parenteral vaccination campaigns took place across the entire city from October to December in 2012 and 2013. The organisational details and results of the vaccination campaigns are explained in detail elsewhere [13,19,20]. Both campaigns reached consecutive vaccination coverage of above 70% leading to the short-term elimination of the rabies virus from N’Djaména [20]. However, the PEP demand remained high even when there were no animal rabies cases (January–October 2014) [13]. To investigate why successful rabies control in the animal sector did not translate to beneficial effects in the human health sector through a lower demand for PEP, we investigated the available epidemiological follow-up data at the laboratory and health facility level.

### 2.2. Laboratory Data on Suspected and Confirmed Animal Rabies Cases

The IRED is the only rabies laboratory in Chad equipped to perform the standard fluorescent antibody test (FAT). In addition to the FAT, samples at the IRED were analysed with the Rapid Immunodiagnostic Test (RIDT) [21]. All positive samples were sent to the Pasteur Institute in Paris for virus isolation by polymerase chain reaction (PCR), to confirm the test results. Rabies surveillance in Chad is based on passive reporting; thus, animals are brought to the IRED on a voluntary basis with no active contact tracing. In most cases, the animal dies or is killed before its submission to the IRED. When an animal is still alive, the rabies laboratory refers the owner to the nearby public veterinary clinic for observation. The IRED charges the owner of the animal 5000 FCFA (8 USD) for rabies diagnostic testing, and there is no charge for feral dogs. The rabies surveillance in N’Djamena continued before, during and after the vaccination campaigns. Some samples from areas outside of N’Djaména were also sent to the IRED for a rabies diagnosis. The present analysis includes cases reported within the time period from June 2012 to end of December 2014, to mirror the data collection period at the health facility level. Information on the animal (vaccination status, location, symptoms observed, and outcome), on the bite victims (number, age, sex, and bite location and severity) and on the history of the bite (time, place, and circumstances) were routinely collected on the diagnostic request sheet. If the test result was positive, the victims were advised to initiate PEP at the Mother and Child Hospital (Hôpital Mère et Enfant), where the vaccine was available free of charge to women and children. Adult male victims were referred to the Central National Reference Hospital (Hôpital Centrale de Reference National).

### 2.3. Health Facility Data on Animal Bite Victims

Data collection on bite cases was performed as in a previous study estimating human deaths from animal bite injuries in N’Djaména [22]. The health facilities for inclusion were identified using the same list as the previous study performed in 2008 [22]. However, not all facilities were still operating, and some new structures were identified during exploratory visits to the districts. In total, 91 facilities were contacted and included in the awareness campaign, representing all the public health centers in N’Djaména, the most frequented private health structures (medical practice) and hospitals (for profit and non-profit), and major pharmacies that had the capability to store the rabies vaccine. The largest private veterinary facility (veterinary practice) was also included. A questionnaire, developed for the study in 2008 [22], was distributed to the facilities, and personnel were asked to complete a form for every bite case presented. The information collected included demographic information about the victim (residence, age, and sex), the nature of the bite wound (severity, number and location), the circumstances of the bite incident (place, provoked/unprovoked, and other victims), the background of the animal (owner status, vaccination status, location, and outcome) and contact with other human health or veterinary structures (referrals from or to other facilities, including the IRED). The source of information related to the biting animal was sometimes the dog owner but could also be the victim or their representative (especially in the case of feral dogs). The decisions on the actions to be taken regarding the animal were primarily made by the owner, but they could also be made by the victim, in the cases of unowned dogs. The questionnaires were collected biweekly by study personnel, with an incentive of 300 FCFA (0.5 EUR) per completed questionnaire provided to the participating health structure. Health facilities were informed about the study in May 2012, and the questionnaires were collected from June 2012 until the end of December 2014. Sixty-one facilities responded with at least one questionnaire (mean of 19.7, median of 4, and range of 1–143; Table 1). This number represented about 30% of the health facilities identified during the study in 2008 [22].

### 2.4. PEP Use and Cost

The WHO recommends including rabies immunoglobulin (RIG) in the PEP protocol for cases with a category III exposure (transdermal injuries or contact of saliva with mucosa) [23]. However, RIG is not available in Chad, and therefore PEP only includes active vaccination with cell culture vaccine (CCV) given according to the intramuscular five-dose Essen regimen [24]. The price of one dose of the human rabies vaccine ranges between 9000 and 12,000 FCFA in N’Djamena, such that a full course of PEP costs 45,000–60,000 CFA (80–100 USD). Adding the costs for wound treatment (antiseptic, antibiotics, or tetanus vaccine), the private costs of lost work time and the transportation to a health facility, the full PEP and bite treatment costs are estimated to be over 90,000 FCFA (160 USD) per case [13]. Our study did not include any follow up of the bite victims; thus, data on the completion rate and the outcome for rabies-exposed people is not available.

### 2.5. Data Analysis

The questionnaire data was double entered and compared using Epi Info, and was then transferred to an Access (Microsoft, Redmond, DS, USA) database. Data collected at the rabies laboratory were entered continuously into an Excel spreadsheet. For both data sets, the analysis was performed with Stata/IC 14. The dog rabies incidence was calculated on the basis of the number of positive dog rabies cases observed in N’Djaména over the study period and the dog population estimates for the city obtained during the two vaccination campaigns, ranging from 24,547 in 2012 to 30,074 in 2013 [19]. The national human population census of 2009 provided by the Chadian national statistical institute for economic and demographic studies (Institut National de la Statistique des Etudes Economiques et Démographiques; INSEED) served as the basis for the calculation of the bite exposure and PEP incidence. Only bite cases reported from N’Djaména (1143) were included for this calculation. To statistically evaluate differences in the incidences (dog rabies cases, dog bites, and PEP use) before and after the mass vaccination intervention, paired *t*-tests were performed. The respective monthly incidences observed from June to December 2012 were compared to the monthly incidences of the period of June to December 2013.

For the analysis of the health facility data, the vaccination status of the biting animals was categorised as “vaccinated” (date of vaccination reported and less than one year before reported bite incident), “vaccination unconfirmed” (missing vaccination date or more than one year before bite incident) or “unvaccinated” (vaccination status reported as unvaccinated or unknown). In addition, each bite case was attributed a rabies-exposure risk variable on the basis of the status and outcome of the animal as drawn from the information in the questionnaire: “high exposure risk” was attributed to animals reported to have disappeared or have been killed after the bite attack; “moderate exposure risk” was attributed to animals with a negative, unclear or out-dated vaccination status, regardless of being under observation or not. Vaccinated animals that bit more than two people were also defined as having a moderate exposure risk because data from the laboratory level showed that the number of victims by case was related to a positive test result. In the cases of no known owner for an animal that was reported to be under observation, the exposure was also considered to be moderate. “No exposure risk” was attributed when the animal had a confirmed vaccination status, had bitten no more than two people, and was placed under observation. The rabies-exposure risk categories, derived from the animal’s status as described above, were compared to the rabies suspicion as noted by the health personnel in the questionnaire (“yes”, “no”, or “do not know”). To evaluate the parameters influencing the PEP recommendation, the respective explanatory variables were coded into categories: the severity of the bite was coded as WHO category II exposure/WHO category III exposure (WHO category I was not observed in this data) [23]; age was coded as adults (>15 years)/children (≤15 years); the number of bites was coded as single bite/multiples bites; the number of victims was coded as single victim/multiple victims. The categories per parameter were then compared by risk ratio (*RR*) analysis with calculation of respective confidence interval (*CI*). A statistical comparison of the PEP recommendations per respective categories was performed by an odds ratio (*OR*) calculation. Regarding the risk of a dog being killed after having inflicted a bite, the 10 districts of N’Djaména were assigned an observed predominant cultural background (Christian/Muslim) and the differences between the two categories were evaluated by a calculation of the *RR*.

### 2.6. Ethical Consideration

The dog rabies mass vaccination intervention was approved and co-funded by the government of Chad. The data on rabid animals and human bite exposure was collected on a routine basis by the rabies laboratory at the IRED. This study was approved by the ministry for higher education in Chad (Letter N°012/PR/PM/MES/SG/DGESRSFP/DRST/012; Date: 31 May 2012). Meetings were held with the mayor of N’Djaména and the district and quarter chiefs in each administrative area who granted permission prior to beginning the study.

## 3. Results

### 3.1. Rabies Diagnostic Results and Respective Case Histories

The awareness campaign before the mass vaccination led to a rapid and considerable increase in the number of rabies-suspicious animals reported to the IRED, from a monthly mean of 1.2 diagnostic requests observed from January to May 2012 (prior to the study period) to 3.6 observed from June to December 2012 (during the study period). Throughout the study period (June 2012–December 2014), a total of 60 rabies-suspect animals were sent to the IRED, of which 46 originated from N’Djamena, 9 originated from other areas in Chad (mostly located very close to N’Djaména), and 2 originated from Cameroon (which borders N’Djaména). In three cases, the sample origin was unknown. Overall, 32 samples tested positive, 25 were negative and 3 were not testable because of a poor sample quality. Table 2 shows the distribution of the test results by species.

In total, 30 (67%) of the submitted dog samples and 2 (33%) of the cat samples were positive. No positive cases were observed in other species. Amongst all animals sent to the IRED, only 10% (five dogs and one cat) were initially put under observation prior to death and subsequent submission to the IRED. In two of those six cases, the animals tested positive for rabies. In most cases, the animal was killed immediately after a bite rather than put under observation (43 cases; 72%). The percentage of positive cases among killed animals was 67% (29 out of 43). The percentage of confirmed rabid animals that were found dead or that died during the observation period was 22% (two out of nine). This observed difference was found to be significant, but with a large confidence interval due to limited sample size (*RR* 3.7; 95% *CI*: 1–13.2; *p* = 0.043). In eight cases, the circumstances of death were not specified.

The majority of animals brought to the IRED were owned (72%), but only 2 out of 32 (6%) owned dogs had a valid vaccination (vaccination <1 year), which was confirmed by a certificate. In one of these two cases, the dog nonetheless tested positive. In six cases, the vaccination status was unconfirmed or out of date and five of these dogs tested positive. Animals other than dogs were all unvaccinated. The most commonly observed symptoms were aggression (88% of cases) and a sudden change of behaviour (40% of cases).

On average, two (min: one; max: six) human bite victims were observed per rabid animal. The proportion of children among the bite victims of confirmed rabies cases was 42% (25 of 59). For rabies-negative cases, the proportion of children among all victims was only 27% (3 out of 11). However, risk ratio analysis showed that this difference was not statistically significant (*RR* 1.4; 95% *CI*: 0.5–3.8; *p* = 0.5)

### 3.2. Reported Bite Cases and Related Animal History at the Health Facility Level

In total, 1203 questionnaires were collected from health facilities during the survey. Three questionnaires were excluded from the data set because the biting animal was a snake and one questionnaire was not completed. The vast majority of the remaining 1199 bite cases were those inflicted by dogs (936 cases; 78%), followed by cats (58 cases; 5%) and monkeys (15 cases; 1.5%). For the remaining 16% (190 cases), information on the animal was missing. A high number of reported victims were children ≤13 years of age (42%).

**Figure 1:**
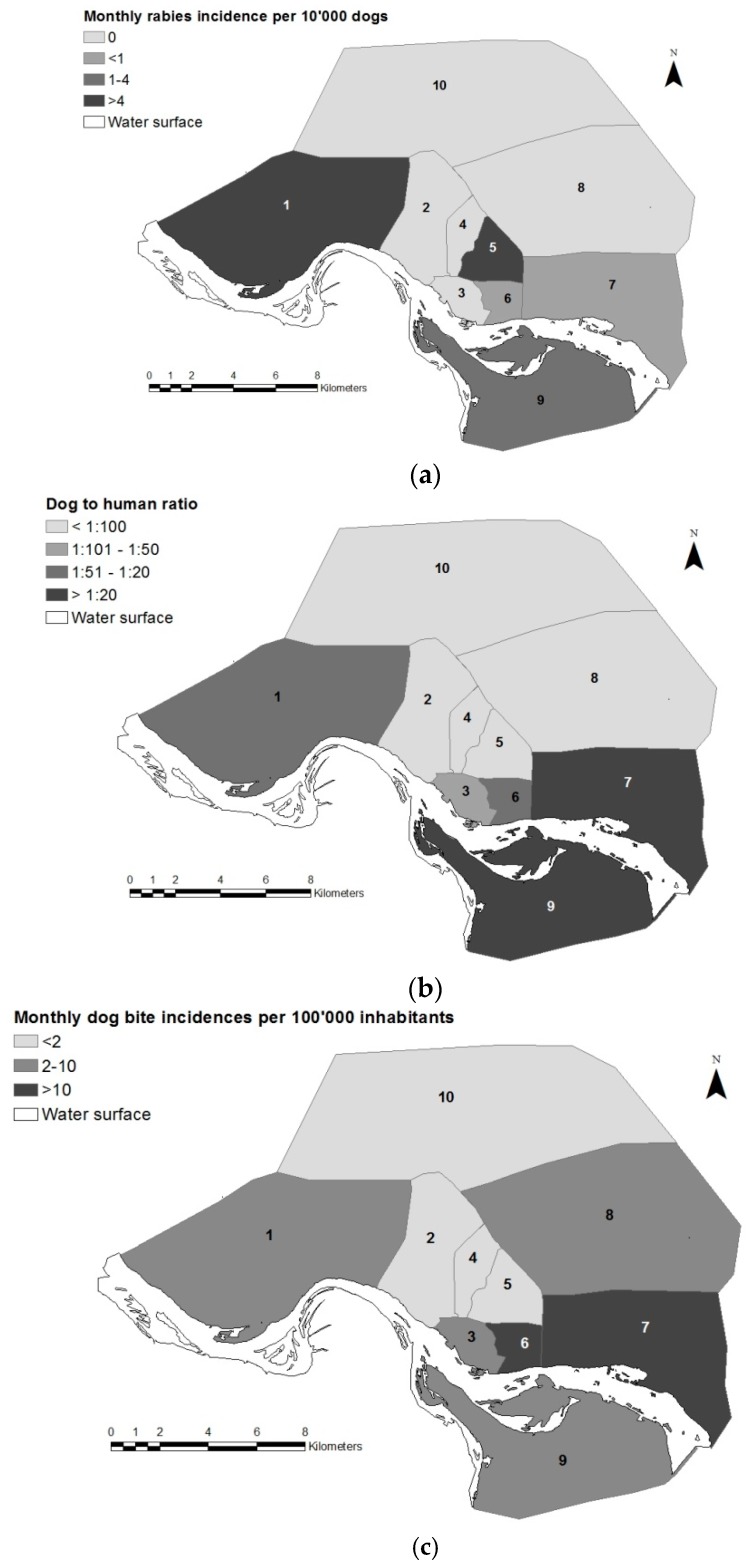
Maps of N’Djaména depicting monthly dog rabies incidences (**a**), dog-to-human ratios (**b**) and monthly dog bite incidences (**c**) observed from June to December 2012. Dog population estimates are based on the results of the vaccination coverage analysis in 2012 published previousely [19]. The human population by district is derived from the population census of 2009 (INSEED). Numbers on the maps indicate the district number.

More than 58% of bite exposures took place at the victim’s home, while an additional 36% occurred very close to the place of residence. Table 3 shows the distribution of reported bite cases and health facilities by district and inhabitants on the basis of the population census of 2009 (INSEED). District 10 was very sparsely represented; only one health facility participated and only 11 bite cases were reported from the entire district (Table 3). There was a low representation of health facilities in this district because of its remote location on the periphery of the town. Additionally, a very low dog population density was reported from this district during the vaccination campaigns [19]. In contrast, districts 6 and 7 had the highest rate of questionnaires per inhabitants (Table 3). This was in accordance with the high density of health facilities and the very high dog-to-human ratio found in these two districts (Figure 1).

District 9 had a similar dog density to district 7, however, like district 10, it was an area at the periphery of the town, where health facilities were not numerous. Figure 1 also shows the monthly incidences of dog rabies and bite exposures by district reported before and during the first vaccination round in 2012 (June–December).

In over 70% of the bite cases, the animal was reported to be put under observation, but in less than one in four of these cases (23.5%) was it noted that the animal was taken to a veterinary facility. A total of 144 cases (15%) stated that the animal was brought to the rabies laboratory at the IRED. However, this number does not correspond to the actual number of animals submitted to the rabies laboratory within the same time period; see above. Moreover, in only 2 out of 72 cases for which the animal was killed and in 1 out of 4 cases for which the animal died was it reported that the carcass was sent to the rabies laboratory. Six dogs were killed, despite a reported confirmed vaccination status. For bite cases reported from districts with a predominantly Muslim background, the animal was 10 times more likely to be killed, compared to those of bite cases occurring in districts with a predominantly Christian background (*RR*: 10.5; 95% *CI*: 6.37–17.36; *p* < 0.0001). The mean number of victims per biting animal observed during the health facility survey was 1.5 (max: 13). In the majority of cases (82%), only one victim was reported.

The highest number of bite victims was reported by pharmacies. Compared to the other types of health facilities involved, hospitals had the highest mean number of reported bite cases, followed by pharmacies and health centers (Table 1).

**Figure 2:**
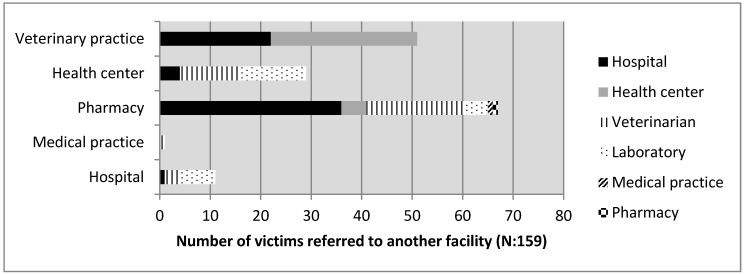
Cases of referral of victims to another health facility.

In total, 161 victims were referred to another facility (Figure 2), and pharmacies were the facilities referring the highest numbers of people to another facility. In 64% of the referrals, the patients were sent to another human health facility, the majority to hospitals (54%), likely because of a shortage of the vaccine or for further wound treatment. In only 36% of referrals were the victims sent to a veterinary facility (28.5% to veterinarians and 7.5% to the IRED). Hospitals and health centers were the facilities that referred the highest numbers of cases to veterinary facilities or to the rabies laboratory at IRED (83% of overall referred cases). The single veterinary facility that participated referred 74% of their bite victims to a hospital or a health center, assumedly because a veterinary facility would not provide the human vaccine.

### 3.3. PEP Treatment Decisions on the Health Facility Level

**Figure 3:**
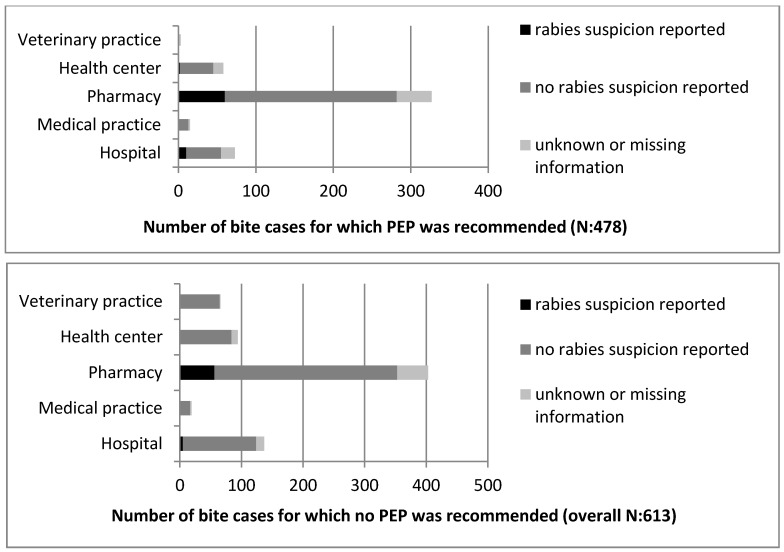
Comparison of post-exposure prophylaxis (PEP) recommendation and reported rabies suspicion by facility type.

The PEP treatment recommendation was not always consistent with the rabies suspicion status reported by the health personnel (Figure 3). In 27% of the bite cases, it was noted that the animal was not suspected for rabies but PEP was recommended. In contrast, 56 patients (0.05%) were not recommended to undergo PEP although the animal was reported as a rabies suspect (Figure 3).

When comparing the rabies exposure risk status defined during analysis (as based on the information available regarding the biting animal), with the suspicion status reported by the health personnel, we found 40 cases in which no suspicion was reported but a high exposure risk was assigned on the basis of the case history. Similarly, when the exposure risk was absent, as assessed by the case history, 54 cases were nonetheless identified by the health personnel as suspected for exposure to a rabid animal.

**Figure 4:**
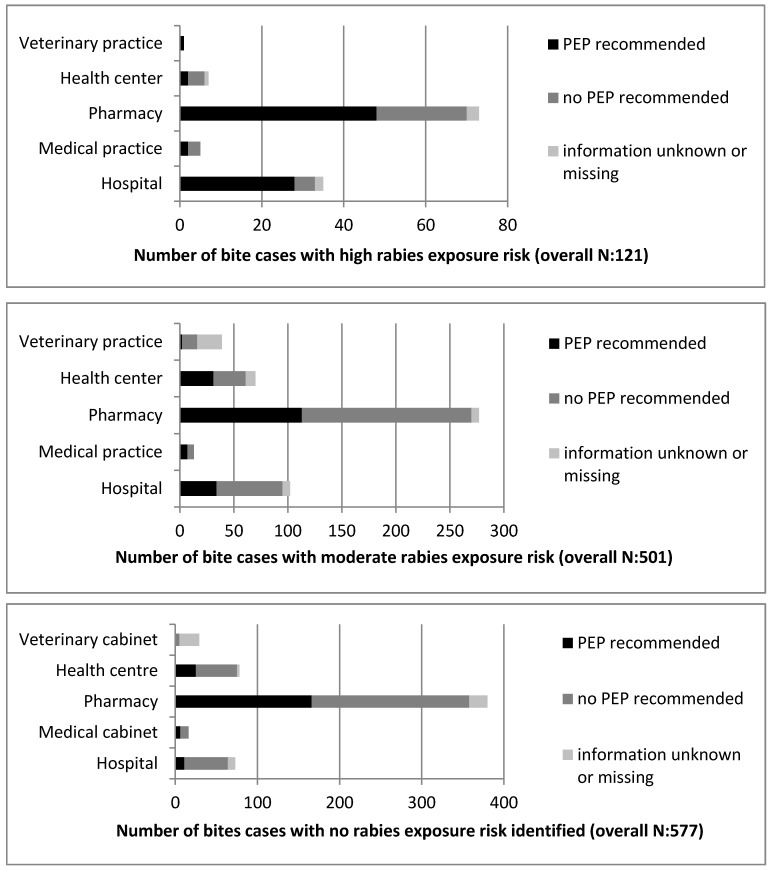
Comparison of post-exposure prophylaxis (PEP) recommendations made by facility type and rabies exposure risk category (as attributed to bite cases on the basis of the animal status).

The comparison of the rabies exposure risk status with the PEP recommendation illustrated even higher discrepancies. Of all the bite cases, including those reported from outside N’Djamé na, with no indication of suspected rabies exposure, 36% (208 out of 577) were recommended to undergo PEP treatment. In 38% (189 out of 487) of the cases for which PEP was recommended, the exposure risk was moderate. Finally, in only 17% (81 out of 487) of the cases for which PEP was recommended did the history of the animal indicate a high exposure risk. Most alarmingly, in 62% (312 out of 501) of all the cases judged to be of moderate exposure risk and in 33% (40 out of 121) of those judged as high exposure risk, the bite victims were not advised to undergo PEP treatment. Detailed results on the PEP recommendations by facility type and the comparison to the rabies exposure risk are presented in Figure 4.

The inconsistencies in the PEP recommendations can be explained by the fact that health personnel were more likely to judge the rabies suspicion according to the severity of the bite inflicted rather than on the vaccination status of the animal. When the wound was deep or the skin was clearly broken, one in five cases was declared as suspicious, and in 50% of these cases, PEP was recommended. In contrast, when the wound was reported as superficial or only a minor scratch, less than 1 in 20 were reported suspicious and 28% of these patients were recommended to undergo PEP. PEP was found to be more likely recommended for WHO category III than category II exposures (*OR*: 0.36; 95% *CI*: 0.28–0.46; *p* < 0.0001). No difference was observed for the PEP recommendation between children and adults (*OR* 1.0065; 95% *CI*: 0.79–1.28; *p* = 0.96) or the number of bites inflicted (single or multiple; *OR*: 1.17, 95% *CI*: 0.9–1.52; *p* = 0.22). There was also no relation observed between the number of victims reported per animal (single or multiple) and the rabies suspicion status defined by the health personnel (*OR*: 0.81; 95% *CI*: 0.51–1.3; *p* = 0.4).

### 3.4. Impact of Mass Dog Vaccination on Dog Rabies Incidence, Animal Bite Incidence and PEP Demand

**Figure 5:**
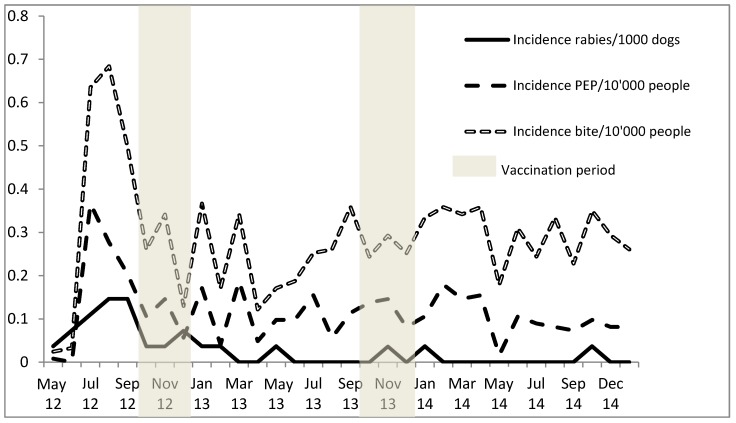
Dynamics of monthly animal rabies, human bite exposure and post-exposure prophylaxis (PEP) demand incidence rates following the dog mass vaccination intervention in N’Djaména.

After the beginning of the vaccination intervention in October 2012, rabies reports from N’Djaména as observed at the IRED dropped steadily (Figure 5). Following the mass vaccination, the monthly animal rabies incidence dropped from 1.1/10,000 dogs, observed prior to the campaign in 2012, to 0.12/10,000 dogs in 2013, and only 0.061/10,000 dogs in 2014. This translates as a reduction from one rabid dog per week in 2012 to only two rabid dogs throughout the whole year in 2014. During the same period of time, the reporting of rabies cases from areas outside of N’Djaména was steady but remained low (only 11 cases over the study period), mainly due to lower public awareness and logistical challenges. Before the vaccination intervention, most rabies cases were reported from the seventh district (Figure 1). After the mass vaccination, rabies cases were absent from districts north of the Chari River for well over a year (February 2013 to October 2014); the only cases observed during this period came from district 9, which lay south of the Chari River.

Figure 5 shows that the incidence of PEP treatment did not decline with the decline of the animal rabies incidence but was instead closely linked to the overall bite incidence rates observed from the health facility data over the study period. For the dog rabies incidence, the paired *t*-test showed a significant difference between the two periods from June to December in 2012 and 2013 (*p* = 0.0063; *t* = 3.8). The difference in the monthly mean PEP incidences over the same period of time was not significant (*p* = 0.1; *t* = 1.4), nor was the difference observed between the monthly mean dog bite incidences (*p* = 0.1; *t* = 1.5).

The proportion of confirmed vaccinated animals among all the bite-inflicting animals observed on the health facility level only increased by 15%, from 47%, observed prior to the start of the vaccination campaign, to 62% by the end of 2014. The percentage of unvaccinated animals decreased from 23% to 11% and cases with an unconfirmed, out-of-date or unknown vaccination status remained stable at around 30%.

## 4. Discussion

The multiple studies undertaken in N’Djaména since 2000 serve as a good example for investigation on the feasibility and description of barriers and shortcomings for rabies control in Sub-Saharan Africa. The most recent work that included a citywide dog mass vaccination and a follow-up of the epidemiological impact has demonstrated the interruption of rabies transmission [20], but it highlights the need for better communication between the veterinary and human health sectors to translate success in dog vaccination to beneficial economic effects in public health [13]. In the absence of dog vaccination, the current PEP use in N’Djaména is inadequate to prevent all dog-mediated human deaths [13]. In this paper, we describe underlying problems that contribute and that should hypothetically be similar in other countries. These factors constitute barriers to the goal of achieving zero human deaths due to dog mediated rabies and they should be addressed early towards the 2030 agenda. The challenges crystallise around the implementation of an integrated, community-based One Health approach for efficient rabies surveillance and control. This can only be achieved through high public participation in control measures (vaccination and surveillance), compulsory dog registration and IBCM training for health and veterinary personnel to ensure the best practice use of PEP.

Our data illustrate the evident fact that the elimination of dog-mediated rabies will not lead to considerably lower bite case incidences, as only a very small proportion of bites are caused by rabid animals. For example, in the present study, the number of bite victims of truly rabid animals recorded at the laboratory constituted only 5% of the overall observed bites in the health facility survey during the same period of time. Therefore, it would be an unnecessary burden on the public health sector to recommend PEP for every bite case. Such extensive use of PEP would even be counterproductive given the current shortage of the vaccine, as it would lead to insufficient PEP for actual rabies-exposed victims.

Our reported numbers for the bite incidence are conservative. Not all victims present to a health facility, as many use traditional forms of treatment [25]. In addition, this study only covered about one-third of the facilities in N’Djaména. Therefore, the actual numbers of bite exposures are believed to have been considerably higher. In general, there were fewer health facilities per inhabitant observed in districts at the periphery of the town compared to the central districts, which could have led to an underrepresentation of cases from the peripheral districts. The socio-economic background also differed between the central (wealthier areas) and peripheral (underprivileged areas) districts; poorer communities might have been less represented in this study. However, N’Djaména is a relatively small town (292 km^2^) with adequate road access and public transportation; therefore, the geographical distance to a respective health facility should have a minor influence on health seeking and PEP accessibility. Most dogs in N’Djaména are owned but are free roaming, which results in high contact between humans and dogs. Nonetheless, notable differences in the bite exposure incidences were observed between different districts. N’Djaména reflects the diverse socio-cultural and socio-economic context of Chad. This background has a significant influence on dog ownership [19]. Districts with a predominantly Muslim background (2nd, 4th and 10th district) have a much lower dog-to-human ratio than areas with a Christian context (1st, 7th and 9th district). Similarly, the dog density is extremely low in the wealthy neighborhoods found in the 2nd district as compared to the very high densities found in the slum areas at the periphery of the town (9th district). Because of this diverse context, dog rabies cases and dog bite exposures are likewise heterogeneously distributed. As the dog population estimates were derived from extensive surveys during the mass vaccination campaigns, and because the number of health facilities per district did not depend on the district cultural background, we are confident that these differences were not a result of underreporting in predominantly Muslim areas.

The study did not include a follow-up of bite patients, and therefore we do not know if victims completed all the PEP doses required. Additionally, we do not know how many cases of human rabies occurred during the study period in N’Djaména, as the disease is not notifiable in Chad. In 2008/2009, the annual number of human rabies cases in N’Djaména was estimated to be seven, and an extrapolation of the mean number of victims per rabid dog registered revealed a huge proportion of possibly exposed people who did not seek PEP [22].

Another study limitation was the lack of follow-up on the observation of biting animals, as the collection of such data on the veterinary level was not included. Regional governmental veterinary institutions in Chad do maintain registries on animal observations, but the results are not regularly reported to higher national levels. In our study, we observed that although, after most exposures, the biting animal was reported to be put under observation, a veterinarian was rarely contacted. This indicates that the owner judged his animal to be alive and well, but that no action was taken to check the animal for signs of rabies by a veterinarian. No legislation on rabies control exists in Chad, other than a short paragraph from 1961 that recommends culling stray dogs. Therefore, the 10 day observation period for an animal that has bitten a person cannot be enforced by law. In our study, animals that died were less likely to be tested positive for rabies at the laboratory level. This has important implications for animal welfare; for instance, dogs accustomed to free-roaming were reported by owners to have died of strangulation while kept on a leash.

The high number of rabies-positive cases among animals with an unconfirmed or out-of-date vaccination status highlights the importance of confirming the owner-reported vaccination status by a certificate. This issue is also highlighted when comparing the proportion of biting animals reported as vaccinated by the health facility survey prior to the mass vaccination campaign, which was 47%, with data from a household survey conducted in 2001, for which the proportion of vaccinated dogs was only 19% [26]. As the negative financial implications (cost of PEP for all bite victims) and social consequences (responsibility for a human death) for owners of a rabid dog are unbearable, some may not accurately represent the vaccination status of their animal. On the other hand, the small increase in the proportion of confirmed vaccinated animals observed over all the reported bite cases during the dog vaccination intervention was concerning, and could have been related to the high likelihood of losing paper-based vaccination certificates [19]. Therefore, to establish an effective IBCM, a unique identification and registration system for all dogs is necessary.

The positive result found in a dog that was certified as vaccinated highlights the need for an observation period even for vaccinated animals. Vaccine failure due to inappropriate storage conditions or the inadequate immune response of the animal can never be excluded. Rabies antibody detection and titration is not yet possible in Chad.

Our findings at the laboratory level indicate that the symptoms of rabies and the abnormal behavior of animals are generally well interpreted. When there was a suspicion of rabies, especially for aggressive dogs, the animal was killed quickly. However, the focus on aggression could mean that the paralytic form of rabies remains undetected in most cases. The IRED is regularly contacted by bite victims seeking PEP treatment who have already killed and disposed of the biting animal. The extremely low number of animals brought to the IRED after being killed or found dead shows that the importance of testing is not adequately perceived. One explanation could be the cost of the diagnostic fee, which is borne by the dog owner. Interaction with victims at the IRED indicated that they usually had little doubt about the symptoms observed in the biting animal and whether they had been truly exposed. This could explain a perception that additional laboratory testing is not necessary.

In addition to cases in which animals that died or were killed were not submitted for testing, there were several cases in which the animal disappeared. This could indicate that the actual number of rabies cases was significantly higher than reported in this study. The observed difference in killing rates between different cultural backgrounds could further point towards an underrepresentation of rabies cases from specific areas of town. However, even when simulating different case detection rates using a rabies transmission meta-population model established for N’Djaména, the outcome of the model, which suggested an interruption of rabies transmission, remained robust [27].

Despite the observed drop in the rabies incidence and the absence of animal rabies cases for nine months following the mass vaccination intervention in N’Djaména, the PEP demand remained largely unaffected by this epidemiological change. The PEP demand was clearly correlated to the overall number of bite exposures observed. The assessment of the rabies risk by health personnel was found to be based on the severity of the bite inflicted. However, scratches are listed as WHO category II exposures and they would therefore also warrant PEP treatment [23]. The influence of the animal status on the rabies risk rating was secondary. Moreover, very few patients were referred by health facilities to a veterinarian for animal observation/advice. All these findings clearly indicate that exchange between health personnel and veterinary workers remains inadequate for IBCM, and should be improved using inter-disciplinary training and communication platforms.

We only included one veterinary facility and this data was insufficient to compare the performance of veterinary facilities with human health facilities. However, the 69 cases of animal bite victims who sought help at the private veterinary practice likely indicate an understanding amongst the public for the link between human and animal health, which provides a basis for the scaling-up of knowledge.

Similarly to other studies, we report a significant number of children among the bite victims [28,29,30]. Also, we found that a high number of bite incidents occurred at home. School-based education and the sensitisation of dog owners regarding rabies and the prevention of dog bites should lead to a decline in bite cases. Raising awareness and knowledge about the rabies risk in communities is an important element to prevent the excessive demand of PEP [31,32] and the unnecessary killing of suspected dogs [33,34] resulting from a fear of rabies. Therefore, a third pillar to establish effective IBCM, along with dog registration and interdisciplinary training, includes community engagement and culturally sensitive education.

## Figures and Tables

**Table 1:** Number of responding health facilities and questionnaires collected.

Facility Type	Health Facilities		Questionnaires		Quest/Facility
	Count	Percent	Count	Percent	
Pharmacy	33	54%	729	61%	22
Veterinary practice	1	2%	69	6%	69
Hospital (public)	6	10%	210	18%	35
Medical practice	6	10%	33	3%	6
Health center (public)	15	25%	154	13%	10
Missing information	N/A	N/A	4	0%	N/A
**Total**	**61**	**100%**	**1199**	**100%**	N/A

**Table 2:** Summary of results of samples received for rabies diagnosis at the Institut de Recherche en Elevage pour le Développement (IRED), by species.

Species	Negative	Positive	No Result	Total
Dog	13	30	2	45
Cat	3	2	1	6
Monkey	6	0	0	6
Sheep	2	0	0	2
Shrew	1	0	0	1
**Total**	**25**	**32**	**3**	**60**

**Table 3:** Number of questionnaires and participating health facilities per district and inhabitants on the basis of the population census of 2009 (INSEED).

District Number	Questionnaires (Q)		Health Facilities (HS)		Population (P) 2009		HS/1000P	Q/1000P
	Count	Percent	Count	Percent	Count	Percent		
1	54	5%	8	13%	72,742	8%	0.11	0.73
2	9	1%	3	5%	36,450	4%	0.08	0.25
3	22	2%	6	10%	38,101	4%	0.16	0.58
4	14	1%	3	5%	72,954	8%	0.04	0.19
5	39	3%	3	5%	102,169	11%	0.03	0.38
6	122	11%	6	10%	43,948	5%	0.14	2.64
7	700	61%	17	28%	221,811	23%	0.08	3.08
8	106	9%	12	20%	185,065	20%	0.06	0.53
9	66	6%	2	3%	75,893	8%	0.03	0.86
10	11	1%	1	2%	98,982	10%	0.01	0.10
**Total (N’Djaména)**	**1143**	**100%**	**61**	**100%**	**948,115**	**100%**	**0.06**	**1.17**
